# Variability in the Initial Costs of Care and One-Year Outcomes of Observation Services

**DOI:** 10.5811/westjem.2015.2.24281

**Published:** 2015-04-10

**Authors:** Ibrahim Abbass

**Affiliations:** University of Texas School of Public Health, Houston, Texas

## Abstract

**Introduction:**

The use of observation units (OUs) following emergency departments (ED) visits as a model of care has increased exponentially in the last decade. About one-third of U.S. hospitals now have OUs within their facilities. While their use is associated with lower costs and comparable level of care compared to inpatient units, there is a wide variation in OUs characteristics and operational procedures. The objective of this research was to explore the variability in the initial costs of care of placing patients with non-specific chest pain in observation units (OUs) and the one-year outcomes.

**Methods:**

The author retrospectively investigated medical insurance claims of 22,962 privately insured patients (2009–2011) admitted to 41 OUs. Outcomes included the one-year chest pain/cardiovascular related costs and primary and secondary outcomes. Primary outcomes included myocardial infarction, congestive heart failure, stroke or cardiac arrest, while secondary outcomes included revascularization procedures, ED revisits for angina pectoris or chest pain and hospitalization due to cardiovascular diseases. The author aggregated the adjusted costs and prevalence rates of outcomes for patients over OUs, and computed the weighted coefficients of variation (WCV) to compare variations across OUs.

**Results:**

There was minimal variability in the initial costs of care (WCV=2.2%), while the author noticed greater variability in the outcomes. Greater variability were associated with the adjusted cardiovascular-related costs of medical services (WCV=17.6%) followed by the adjusted prevalence odds ratio of patients experiencing primary outcomes (WCV=16.3%) and secondary outcomes (WCV=10%).

**Conclusion:**

Higher variability in the outcomes suggests the need for more standardization of the observation services for chest pain patients.

## INTRODUCTION

The use of observation units (OUs) following emergency department (ED) visits as a model of care has increased exponentially in the last decade.^1^,^2^ It is estimated that one-third of U.S. hospitals have OUs within their facilities.^3^ While their use is associated with lower costs and comparable level of care compared to inpatient units,^4^–^6^,^19^ there is a wide variation in OUs characteristics and operational procedures.^7^,^8^ Ross and colleagues have listed four major models of OUs in U.S. hospitals.^8^ The differences that characterize these models lie within whether they are protocol driven and/or on whether care is provided at dedicated units.^8^ While two-thirds of hospitals do not have dedicated OUs to observe patients, these hospitals provide observation services to patients in unstructured units that may include any bed within their facilities.^2^,^9^ The majority of hospitals that have dedicated OUs lack protocols or disease-specific guidelines.^8^ Protocol-driven OUs were demonstrated to have lower length of stay and better outcomes compared to other models.^8^ Given the variability in the structure, model and operations of OUs in the U.S., the objective of this study was to explore the variability in the input (initial costs of care) and outputs (one-year outcomes) across OUs. Analysis was limited to patients admitted to OUs due to non-specific chest pain as it is the most cited reason for ED visits among adult population in the U.S.^10^–^12^ and also to limit variability in costs and outcomes imposed by the prognostic characteristics of different diseases.

## METHODS

### Study Design and Data Sources

This was a retrospective cohort study that included patients who had observation services between January 2009 and December 2011 following ED visits for non-specific chest pain (ICD9 = 786.5, 786.50 and 786.59). The author extracted data from BlueCross BlueShield of Texas (BCBS-TX) with preferred provider organization (PPO) and PPO+ plans only. Patients in other plans were excluded due to contractual agreements with the providers or for lacking complete claims of their enrollees. Observation services were defined, per Medicare Claims Processing Manual,^13^ as using a combination of a revenue code (0762, 0760) and Healthcare Common Procedure Coding System classification (HCPCS) code of G0378 (observation service per hour) and G0379 (referral to observation). The author performed costs, outcomes and risk adjustments at the patient’s level and aggregated the averages at the OU’s level.

### Sampling

Patients who were between 18 and 63 years of age and had one year of continuous insurance enrollment prior to and after the ED visit were included in the study. Claims filed in the year prior to the ED visit were used to identify patients’ comorbidities and calculate their risk scores. Outcomes of OUs admission were identified using the claims incurred in the year following the ED visit. Patients were then linked to OUs using the servicing provider identification number (SPID) associated with observation services (G0378 and G0379) on facility claims. Patients who had more than one SPID were excluded. Patients who were subsequently admitted to inpatient units were also excluded. To secure enough representations of patients within each OU, OUs that had less than 30 patients in the final sample were excluded from further analysis.

### Cost of Care

The study took the payer’s perspective in defining costs, which represented the allowed amount paid by the insurer to providers for the rendered services. The initial costs of care included all the medical and professional services incurred between the ED admission and OU discharge dates. All costs were adjusted for inflation to 2012 equivalent cost using the medical inflation factor published by the Bureau of Labor Statistics.

### Outcomes

The author evaluated the effectiveness of OUs using a composite of primary and secondary outcomes that was previously used.^5^,^6^ The primary outcomes included the first occurrence of myocardial infarction, congestive heart failure, stroke, and cardiac arrest. Secondary outcomes included subsequent one-year use of an ED for nonspecific chest pain or angina pectoris, hospitalization due to circulatory disorders, or revascularization procedures specifically percutaneous transluminal coronary angioplasty (PTCA), and coronary artery bypass graft (CABG). The prevalence rates of primary and secondary outcomes across OUs were calculated. Outcomes also included the inpatient, outpatient, and professional costs related to chest pain or cardiovascular diseases incurred in one year following initial OU discharge.

### Clinical Risk Adjustment

Costs and outcomes of OUs discharge are contingent on the clinical condition of the admitted patients and their level of risk. To mitigate potential confounding and bias effect, the author used two methods to account for the different case-mix of patients across OUs. In the first method, patients’ risk scores were calculated using the Adjusted Clinical Group (ACG) software. Scores created by the ACG software represents the burden of illness on patients and using them as a measure of patients comorbidities have been validated and used for similar purposes in many studies.^13^–^16^ The average risk score for the sample population is calibrated to one and patients whose scores are greater than one are at higher risk of incurring more medical care next year. Data required for risk score calculation include patients’ age, gender, up to 10 diagnoses per claim, revenue codes, place of treatment, and total cost of claims filed one year prior to the ED visits. Second, comorbidities that could confound the results of the analysis were identified and adjusted for. This included cardiovascular-related disorders, cardiac procedures, and other conditions that are highly associated with ED visits for chest pain (Table). Diagnoses at discharge and ambulance services use for transport to the ED were used as proxies of urgency of patients’ condition during their ED visits. Details on the codes used to identify comorbidities are provided in the supplementary appendix (Appendix). The author included both the risk scores and patients’ comorbidities in the statistical models.

## ANALYSIS

The mean, median, and frequencies were used to summarize continuous and categorical variables (Table). Differences in patients’ baseline demographics and clinical characteristics across OUs were tested using multivariate analysis of variance (MANOVA) for continuous variables and the randomization test of independence for categorical variables with Monte Carlo simulation with 100,000 replications. The author calculated the unadjusted prevalence rates of primary and secondary outcomes in each OU by dividing the number of patients who experienced outcomes over the total number of patients at each OU. The adjusted prevalence rates of primary and secondary outcomes (prevalence odds ratios) were calculated using two logit models that incorporated patients’ age, gender, comorbidities, and risk scores. The averages of the estimated prevalence odds ratios were then computed for each OU.

The author computed the unadjusted median costs of initial care at OU and the one year costs of chest pain and cardiovascular diseases for each OU by summing the total costs for each patient and then taking the median cost for each OU. Both costs were then adjusted for patients’ age, gender, comorbidities, and risk scores using quantile regressions at the patients’ level. The averages of the predicted costs were then aggregated over OUs.

The variability in the unadjusted and adjusted prevalence of outcomes and costs were examined using the coefficients of variation, which is calculated by dividing the standard deviation by the mean. An increase in the coefficient represents an increase in the variability across OUs. The coefficient of variation is a standard statistical test that is used to compare the variability of factors with different measurement units. Coefficients of variation were then weighted using the number of patients seen at each OU to account for the unbalanced distribution of patients clustered within OUs (range: [30–254). The author conducted the study using SAS 9.3^16^ for data management, and Stata13.1^17^ for statistical analysis.

## RESULTS

### Population Sample

**Figure 1:**
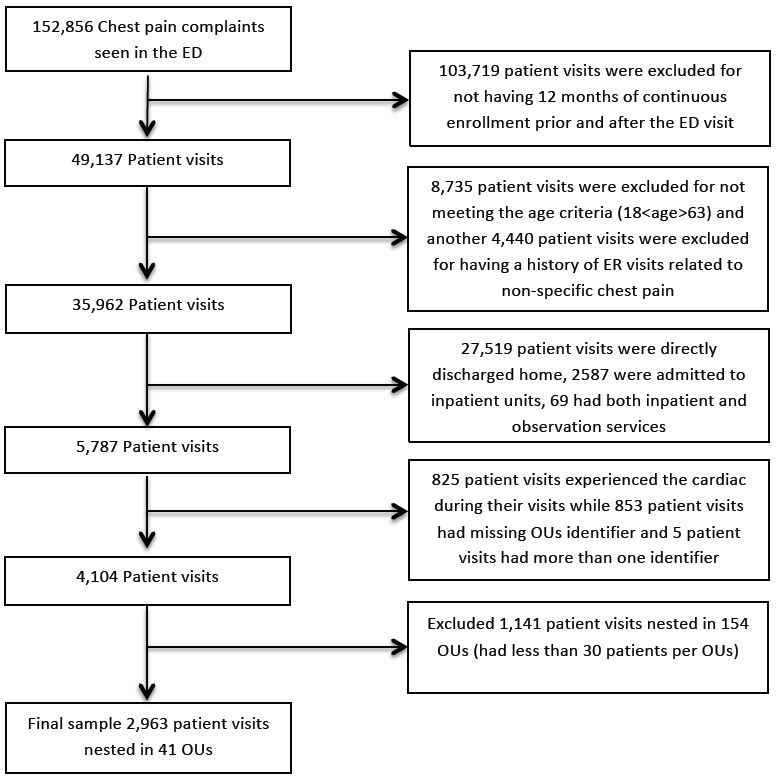
Methodology used to extract the final study sample. *ED*, emergency department; *ER*, emergency room; *OU*, observation units

Figure 1 depicts the methodology that was employed to extract the final study sample. In total, there were 152,856 patient visits to the ED for which the primary complaint was non-specific chest pain. The author excluded 103,719 and 8,735 patient visits for not meeting the continuous enrollment and age criteria respectively. Another 4,440 patients were excluded for having prior ED visits related to chest pain. Patients who were directly discharged home (n=27,519), admitted to inpatient units (n=2,587), had both inpatient and observation admissions (n=69), or experienced cardiac outcomes during their visits (n=825) were excluded. Finally, patients who had missing SPID (n=853) associated with observation services codes or had duplicated OU SPID (n=5) were excluded. This concluded a sample of 4,104 patients who were nested within 195 OUs. Finally, the author excluded OUs with less than 30 patients from further analysis. Thus, the final sample included 2,963 patients nested in 41 OUs. The median number of patients per OU was 56 (range: 30 to 242 patients).

### Patients Baseline Characteristics

Demographic, risk scores, and patients characteristics for the sample population is depicted in the Table. The average age of the sample was 49.8 years old with a majority of females (59%). Patients nested across different OUs had statistically significant differences in their age, gender, risk scores, and clinical comorbidities in chronic rheumatic heart diseases, diseases of veins, lymphatics and other diseases of circulatory systems, diabetes mellitus, dyslipidemia, mental disorders, and ambulance use.

### Outcomes

Within one year of their discharge from OUs, 126 (4.3%) patients experienced a total of 159 primary outcomes, and 302 (10.2%) experienced a total of 434 secondary outcomes. The proportion of patients who experienced primary outcomes included 0.88% (n=26) for MI, 1.55% (n=46) for CHF, 2.13% (n=63) stroke and 0.81% (n=24) cardiac arrest. In contrast, 2.06% (n=61) had revascularization procedures, 7.15% (n=212) went to the ED again within a year for a total of 271 unique visits and 2.8% (n=83) had 101 hospitalization events related to chest pain and cardiovascular related diseases. The unadjusted median cost of an ED episode across all OUs was $5,328 (5^th^ percentile=$3,016, 95^th^ percentile=$10,113), and after adjusting for patients’ differences the median cost went down to $4,838 ($4,646; $6,516). In contrast, the one-year median cost of cardiovascular-related medical services was $238 ($3; $1,694) compared to an unadjusted median costs of $271 ($0; $11,366).

### Coefficients of Variation

**Figure 2:**
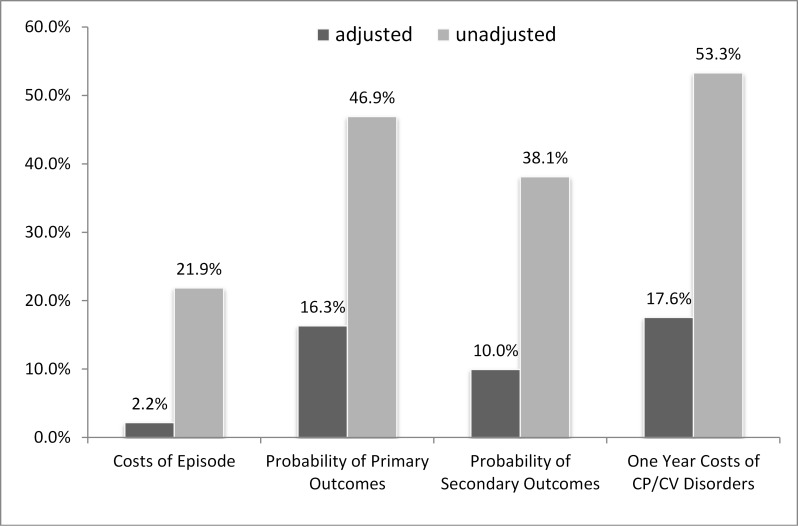
Weighted coefficients of variation demonstrating high variability in the unadjusted costs of initial care and all outcomes. *CP*, chest pain; *CV*, cardiovascular

The weighted coefficients of variation (WCV), as illustrated in Figure 2, demonstrated high variability in the unadjusted costs of initial care and all outcomes. The adjusted weighted coefficients of variation, however, exhibited less variability. A minimal variation (WCV=2.2%) was observed in the initial costs of care while higher variability were observed in the outcomes even after adjustment. The most pronounced variability were associated with the adjusted chest pain/cardiovascular related costs of medical services (WCV=17.6%) followed by the adjusted OR of patients experiencing primary outcomes (WCV=16.3%) and secondary outcomes (WCV=10.0%). Variability in the outcomes was relatively low even though it was higher when compared to the variability of the initial costs of care.

## DISCUSSION

In perfect situations, we expect variability in the inputs to relatively match the variability in the outputs. In this study, input represented the initial costs incurred during patients’ visits to the ED and subsequent admission to OUs. Outputs, on the other hand, were the outcomes that occurred one year after OU admission. The results of this study demonstrate that the variability in the input, after adjusting for patients’ baseline differences across OUs was rather minimal (WCV=2.2%), while the variability in outputs (outcomes) were relatively higher compared to the variability of input (WCV from 10.0%–17.6%). The little variability observed in the initial costs of care is not surprising giving the fact it is governed by payment policies and contractual agreements for the rendered services between the insurer and the different OUs. In contrast, the variability in the outcomes even after adjusting for baseline differences were 7.4, 4.6, and 8 times greater for the primary outcomes, secondary outcomes, and the one-year chest pain/cardiovascular-related costs. Having little variability in the initial costs of care across all OUs does not necessarily imply that all OUs allocate costs in the same manner. Rather, OUs will have different protocols or approaches and allocate services differently. This may imply that the insurer is doing well in reducing variability toward paying for medical services for this specific condition, as they are supposed to do. However, the greater variability in the outcomes, compared to the initial costs of care, might be reflecting the differences in OU models and the variation in the implemented procedures and protocols to manage patients with chest pain within these OUs. According to Ross and colleagues, the majority of OUs lack standardized protocols leaving the provided care at the discretion of the treating physicians.^8^ With that, variations in the employed approaches to treat patients are more likely to yield variant outcomes. Even if care was provided in protocol-driven OUs, the variation between different OUs protocols will more likely yield different outcomes as well. In contrast, protocol-driven OUs have operational guidelines that delineate the inclusion/exclusion criteria, the required staffing and disease-specific guidelines with more focus on quality measurements to ensure better and consistent outcomes.^8^

Even though variations in the outcomes were relatively low, the findings of this study indicate that there is still an opportunity for more savings if payment policies have incorporated outcomes measures as part of the payment schemes. Ross and colleagues proposed establishing different payment schemes that will reimburse OUs according to the model of care.^8^

If payment revision is to be established, then it might be more relevant to base these revisions on quality measures. The results of this study also signify the need to examine the source of variation in the implemented approaches across OUs to investigate best practices in managing patients with non-specific chest pain.

## LIMITATIONS

This study has several potential limitations. First, there might be other underlying factors that drove the variability in outcomes, which were not adjusted for in the logit and quantile models. Nevertheless, factors included in both models reduced the variability of the initial cost of care from 21.9% to only 2.2%. Thus, these models, holding all variables constant, should produce relatively similar variability in the outcomes assuming the absence of other confounders that selectively affect the outcomes but not the clinical condition of patients at the time of OUs admission. Second, market-related factors and regional differences might contribute to the variability of costs and outcomes. While geographic variations across OUs might exist, the purpose of this study was to explore the degree and not the source of variation in costs and outcomes. Third, in calculating the initial costs of care, some claims for services rendered outside the ED/OU are included. The included claims, however, are small as 98.6% of evaluation and management costs, using the Berenson-Eggers Type of Service (BETOS) classification system,^18^ were incurred due to ED visits and consultation while the rest were due to specialists and office-based visits. Finally, the analysis was limited to patients placed in OUs following ED visits due to chest pain only. Thus, the results are not generalizable to OUs stays attributed to other disease conditions. Further studies are needed to examine whether the trends observed in this study hold using broader population.

## CONCLUSION

Variability in the initial costs of care across the different OUs was minimal while greater variability in the outcomes was detected. The results of the study support the need for standardizing observation services for chest pain patients.

## Supplementary Information



## Figures and Tables

**Table:** Baseline characteristics of the population sample using mean, median, and frequencies to summarize continuous and categorical variables.

	Patients’ characteristics (mean, standard deviation, median, frequencies)	p-value§
Age (mean ± STD)	49.8 ± 8.3	<0.001
Gender (male)*	41% (1542)	0.023
Risk score (median)	0.42	<0.001
Chronic rheumatic heart disease (n)	0.8% (30)	<0.001
Hypertensive disease	53.6% (2027)	0.137
Ischemic heart disease	13.7% (520)	0.525
Diseases of pulmonary circulation	1.2% (44)	0.540
Other forms of heart disease†	16.9% (639)	0.790
Cerebrovascular disease	4.3% (164)	0.484
Diseases of arteries, arterioles, and capillaries	3.4% (128)	0.337
Diseases of veins and lymphatics, and other diseases of circulatory system	7.3% (278)	0.027
Diabetes mellitus	17.4% (657)	0.002
Dyslipidemia	46.6% (1764)	<0.001
Diseases of the digestive system	41% (1553)	0.773
Mental disorders	32.4% (1227)	0.006
Diseases of the respiratory system	54.7% (2070)	0.002
Coronary artery bypass grafting	0.5% (18)	0.577
Percutaneous transluminal coronary angioplasty	2.8% (106)	0.110
Used ambulance to reach emergency department	13.7% (517)	<0.001
Diagnosis at discharge: ill-defined	98.4% (3726)	0.602
Diagnosis at discharge: circulatory	0.7% (26)	0.431
Diagnosis at discharge: others	0.9% (33)	0.034

§ P-value indicates whether patients’ baseline demographics and clinical characteristics across OUs are similar or dissimilar.

† Other heart diseases include pericarditis, endocarditis, cardiomyopathy, conduction disorders, dysrhythmia, heart failure and complications of heart diseases.

* Percentages above are the proportion of identified comorbidities over the total number of patient visits in each group.

** Numbers in parentheses are the numbers of patients with the corresponding condition.
