# Supplementary material for: Variability in the Initial Costs of Care and One-Year Outcomes of Observation Services
Source: West J Emerg Med. 2015 Apr 10;16(3):395–400. doi: 10.5811/westjem.2015.2.24281 (PMC4427210; doi:10.5811/westjem.2015.2.24281)
Supplement: Supplementary file 1 [file wjem-16-395-s001.pdf]

**Disease**	Codes
**Current myocardial infarction**	(ICD9 = 410.xx)
**congestive heart failure**	(ICD9 = 428.xx)
**Stroke**	(ICD9 = 430.xx, 431.xx, 432.xx, 434.xx, 435.xx, 436.xx)
**Cardiac arrest**	(ICD9=427.5x)
**Chronic rheumatic heart disease**	(ICD9=393.xx–398.xx)
**Hypertensive disease**	(ICD9 = 401.xx – 405.xx)
**Ischemic heart disease**	(ICD9 = 410.xx – 414.xx)
**Diseases of pulmonary circulation**	(ICD9 = 415.xx–417.xx)
**Cerebrovascular disease**	(ICD9 = 430.xx – 438.xx)
**Diseases of arteries, arterioles, and capillaries**	(ICD9 = 440.xx – 448.xx)
**Diseases of veins and lymphatics, and other diseases of circulatory system**	(ICD9 = 451.xx – 459.xx)
**Diabetes Mellitus**	(ICD9=250.xx)
**Dyslipidemia**	(ICD9 = 272.xx)
**Coronary artery bypass grafting**	(CPT = 33510 - 33519, 33521-33523, 33533-33536)
**Percutaneous transluminal coronary angioplasty**	(CPT = 92980 - 92984)
**Mental disorders**	(ICD9 = 290.xx–319.xx)
**Digestive disorders**	(ICD9 = 520.xx–579.xx)
**Respiratory disorders**	(ICD9 = 460.xx–519.xx)
